# *In Vivo* Evaluation of PAX6 Overexpression and NMDA Cytotoxicity to Stimulate Proliferation in the Mouse Retina

**DOI:** 10.1038/s41598-018-35884-5

**Published:** 2018-12-07

**Authors:** Ehsan Ranaei Pirmardan, Zahra-Soheila Soheili, Shahram Samiei, Hamid Ahmadieh, Seyed Javad Mowla, Marzieh Naseri, Narsis Daftarian

**Affiliations:** 1 Department of Molecular Genetics, Faculty of Biological Sciences, Tarbiat Modares University, Tehran, Iran; 2 Department of Molecular Medicine, Institute of Medical Biotechnology, National Institute of Genetic Engineering and Biotechnology, Tehran, Iran; 3 Blood Transfusion Research Center, High Institute for Research and Education in Transfusion Medicine, Tehran, Iran; 4 Ophthalmic Research Center, Shahid Beheshti University of Medical Sciences, Tehran, Iran; 5 Department of Molecular Medicine, Faculty of Advanced Technology, Iran University of Medical Sciences, Tehran, Iran

## Abstract

Retinal degenerative diseases, due to the lack of regeneration systems and self-renewable cells, often lead to visual impairment. Pax6 is a pleiotropic transcription factor and its expression level determines self-renewal status or differentiation of retinal cells. Here, we investigated the fate of simultaneous induction of retinal ganglion cell death and Pax6 overexpression in retro-differentiation of retinal cells and their commitment to re-enter into the cell cycle. Induction of acute retinal ganglion cell death and generation of mouse experimental model was performed by N-methyl D-aspartic acid (NMDA) injection. Recombinant AAV2 virus harboring PAX6 cDNA and reporter gene was injected into untreated and model mouse eyes. Histological analyses, including IHC and retinal flatmounts immunostaining were performed. The number of Ki67+ cells was clearly increased in model mice, presumably due to NMDA treatment and regardless of Pax6 over-expression. Unlike previous studies, Ki67+ cells were found in GCL layer and interestingly ONL cells expressed Sox2 stemness marker after NMDA cytotoxicity. The potential of retinal cells for robust Ki67 expression, after injury, and expression of Sox2, confirmed their intrinsic plasticity and made a vivid prospect for retinal regenerative medicine.

## Introduction

In the human retina, due to the lack of regeneration systems and self-renewable cells, degenerative diseases often lead to a visual impairment. The vertebrate neural retina composed of six types of differentiated neurons and one type of glial cells and all of them originate in a conserved order from retinal progenitor cells (RPCs). While non-mammalian vertebrates due to the presence of RPCs, throughout their lives, have a remarkable ability to replace damaged neurons, in mammalians we have no significant retinal neurogenic source^1^. Our understanding of many aspects of RPCs is limited, however, because of strong relationship between cell cycle and retinal development^2,3^, it is reasonable to hypothesize that if we can stimulate cells to reenter to cell cycle, with respect to common ancestor, maybe all retina cell types regenerate after degenerations.

Although there are no reports on *de novo* neurogenesis in mammalian adults, but characterization of muller glia as intrinsic retinal stem cells^4^ and some findings about retinal cells plasticity^5,6^ can be indicative of inherent potential of retinal cells for proliferation and regeneration. Furthermore, it is reported that after induction of retinal degeneration, some cells reenter to the cell cycle^7^. Ooto and his colleagues showed neural regeneration after acute neurotoxic injury mediated by N-methyl D-aspartate (NMDA) in the adult mammalian retina^8^. Also, it has been shown some growth factors such as EGF can enforce retinal cells to proliferate^9^.

In recent years, successful reprogramming of adult cells by ancestral transcription factors was a milestone in cellular biology^10–14^. In reprogramming studies, the main goal is perturbing gene regulatory network (GRN) to achieve intended phenotype. In these strategies transcription factors are from the effective and convenient choices. The Pax6 transcription factor resides at the top of the genetic hierarchy controlling development and morphogenesis of the eye and is crucial for the development of the central nervous system (CNS), nose, pancreas, and pituitary gland^15,16^. This factor is one of the indicators of RPCs. Its expression level is a determinative factor in self-renewal status or differentiation of retinal cells^17^. It has been shown that after retinal injuries in mice, some Pax6 positive cells migrate from inner nuclear layer (INL) to outer nuclear layer (ONL); However this migration does not lead to proliferation and differentiation dislike some lower vertebrates^18^.

Here, we investigated perturbing GRN in inner retina layers with PAX6 overexpression, mediated by adeno-associated virus serotype-2 (AAV-2), and induction of retinal ganglion cell death to analyze expression of cell cycle marker in the mouse retina.

## Results

### Induction of retinal damage

**Figure 1:**
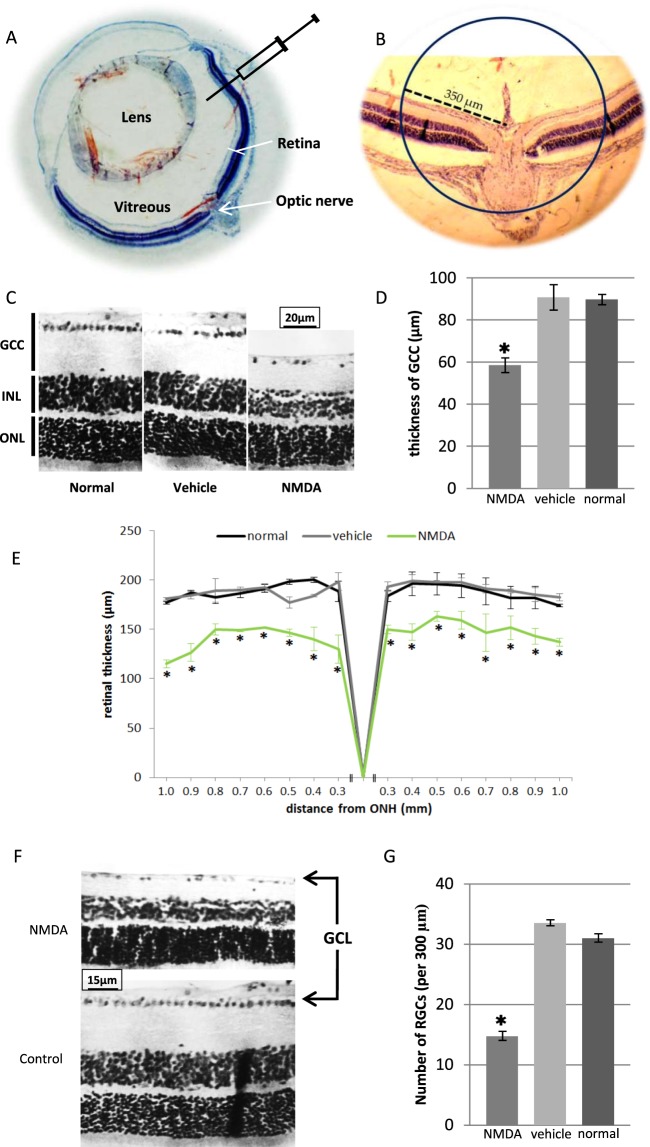
Generation of mouse experimental model by NMDA excitotoxic amino acid. (**A**) Schematic illustration of intravitreal injection of NMDA; All injections were performed under ora serrata by glass needle. (**B**) For evaluation of mice model, analyses were performed on a distinct distance of optic nerve. (**C**,**D**) Histological analyses of GCC thickness 7 days post injection. Data significantly decreased in NMDA samples versus vehicle treated and normal mice. (**E**) Quantitative spider plot analysis depicting significant decrease in retinal thickness in NMDA samples. (**F**,**G**) Histological analysis of number of ganglion cells 7 days post injection. Data significantly decreased in NMDA samples versus vehicle treated and normal mice. GCC: ganglion cell complex; ONL: outer nuclear layer; INL: inner nuclear layer; GCL: ganglion cell layer; ONH: optic nerve head. (*P < 0.05 versus other groups, error bar: means ± SD).

NMDA-induced RGC damage is a reliable method to generate experimental model. Generation of this model characterizes by rapid RGCs loss followed by gradual reduction in ganglion cell complex thickness (GCC: retinal nerve fiber layer, ganglion cell layer, and inner plexiform layer). The thickness maps and the quantitative thickness values of retina and GCC showed thickness changes in the NMDA-treated mice when compared with normal and vehicle-treated mice (Fig. 1C–E). ONL thickness was not affected, but INL thickness showed small changes (see Supplementary Fig. S1). The number of retinal ganglion cells was significantly decreased in treated mice (Fig. 1F,G). Normal and vehicle-treated mice were similar in all parameters.

### Analysis of Ki67 and Pax6 expression in normal mouse retina

**Figure 2:**
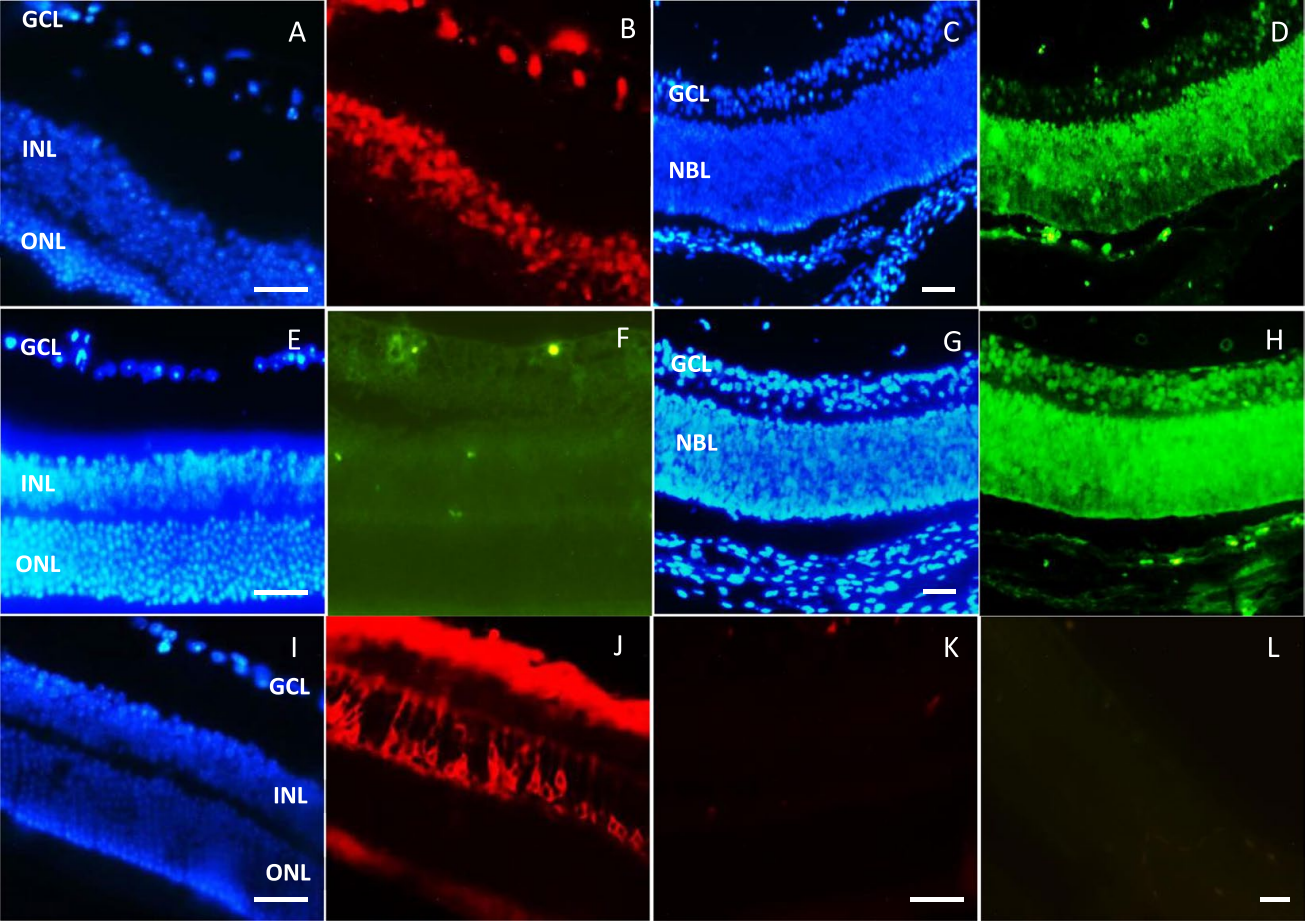
Immunohistochemistry on normal mouse retina for Pax6, Ki67 and PKCα factors. (**A**,**B**) Pax6 expression in adult mouse retina; Pax6 was expressed in GCL layer and inner region of INL. (**C**,**D**) Pax6 expression in newborn (P1) mouse retina; Pax6 was expressed in all retinal layers but its expression was decreased in outer retina layer. (**E**,**F**) Ki67 expression in adult mouse retina; Ki67 was not detected in adult mouse retina. (**G**,**H**) Ki67 expression in newborn (P1) mouse retina; Ki67 was expressed in all retinal layers. (**I**,**J**) PKCα expression in adult mouse retina; PKCα was expressed in GCL layer and some cell types of INL. (**K**,**L**) Negative controls of immunofluorescences for Rhodamine and FITC-conjugated antibodies (no 1^st^ ab.). ONL: outer nuclear layer; INL: inner nuclear layer; GCL: ganglion cell layer; NBL: neuroblastic layer. Scale bar: 50 µm.

Prior to the transduction of the retina, we compared Ki67, Pax6 and PKC expression in uninjected newborn and adult mouse retina (Fig. 2). Pax6 expression was detected in all the retinal layers just after mouse birth. Its expression, as it is shown in Fig. 2C,D, determined a gradient decrease from GCL to ONL. In adult mouse, Pax6 expression is limited to inner cells of INL and GCL cells (Fig. 2A,B).

Ki67 has no expression in adult mouse retina (Fig. 2E,F), while in newborn mouse retina all of the retinal cells expressed Ki67, clearly (Fig. 2G,H). PKCα, as serine/threonine protein kinases, is a cytoplasmic marker in bipolar and ganglion cells. Figure 2I,J, shows that PKCα is expressed in outer region of INL and ganglion cells.

### Infection of mouse retina with AAV-2

**Figure 3:**
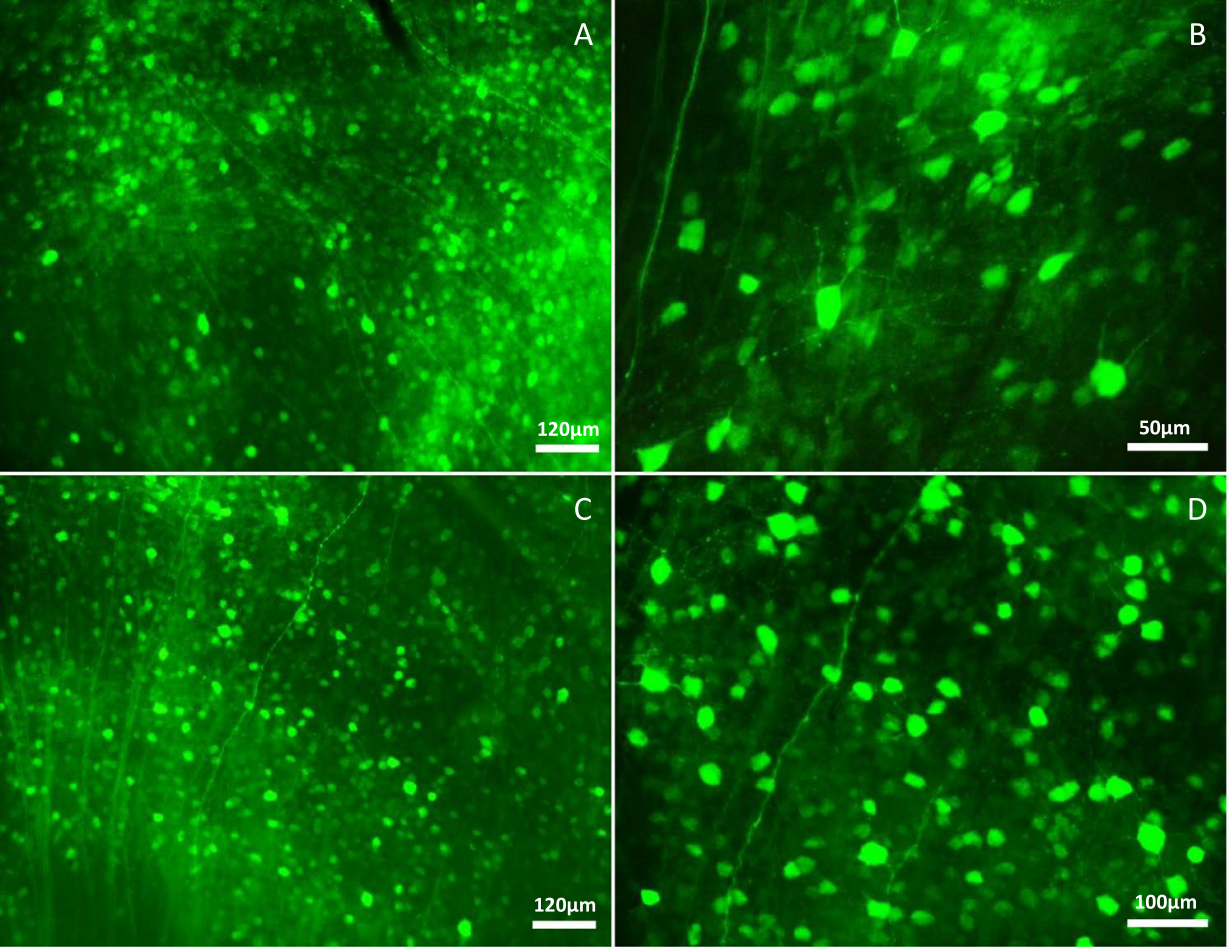
Efficient EGFP expression in whole-mount retina following intravitreal injection of AAV2 viruses (after 3 weeks). (**A**,**B**) EGFP expression following injection of AAV2/EGFP viruses in different magnifications. (**D**,**E**) EGFP expression following injection of AAV2/PAX6-EGFP viruses in different magnifications. Images revealed high infection rate of the recruited AAV preparation, which is criterion for AAV grade and efficiency in transduction. (in all experiments n > 5).

The most widely used vectors for ocular gene delivery are based on adeno-associated virus (AAV), because they elicit minimal immune responses and mediate long-term expression in a variety of non-dividing retinal cell types. Here, we used AAV serotype-2 for PAX6 delivery to the mouse retina. Viral titration was about 10^12^ genomic particles/ml and 10^8^ infectious particles/ml. Radiant green cells infected with AAV2-EGFP viruses represented successful infection in experiments (Fig. 3). For *in vivo* retinal infection, concentration and purification of AAV paticles are the major concerns and a little decline in MOI leads to very significant decrease in infection rate^19,20^.

### Evaluation of Pax6 overexpression in normal and model mice

**Figure 4:**
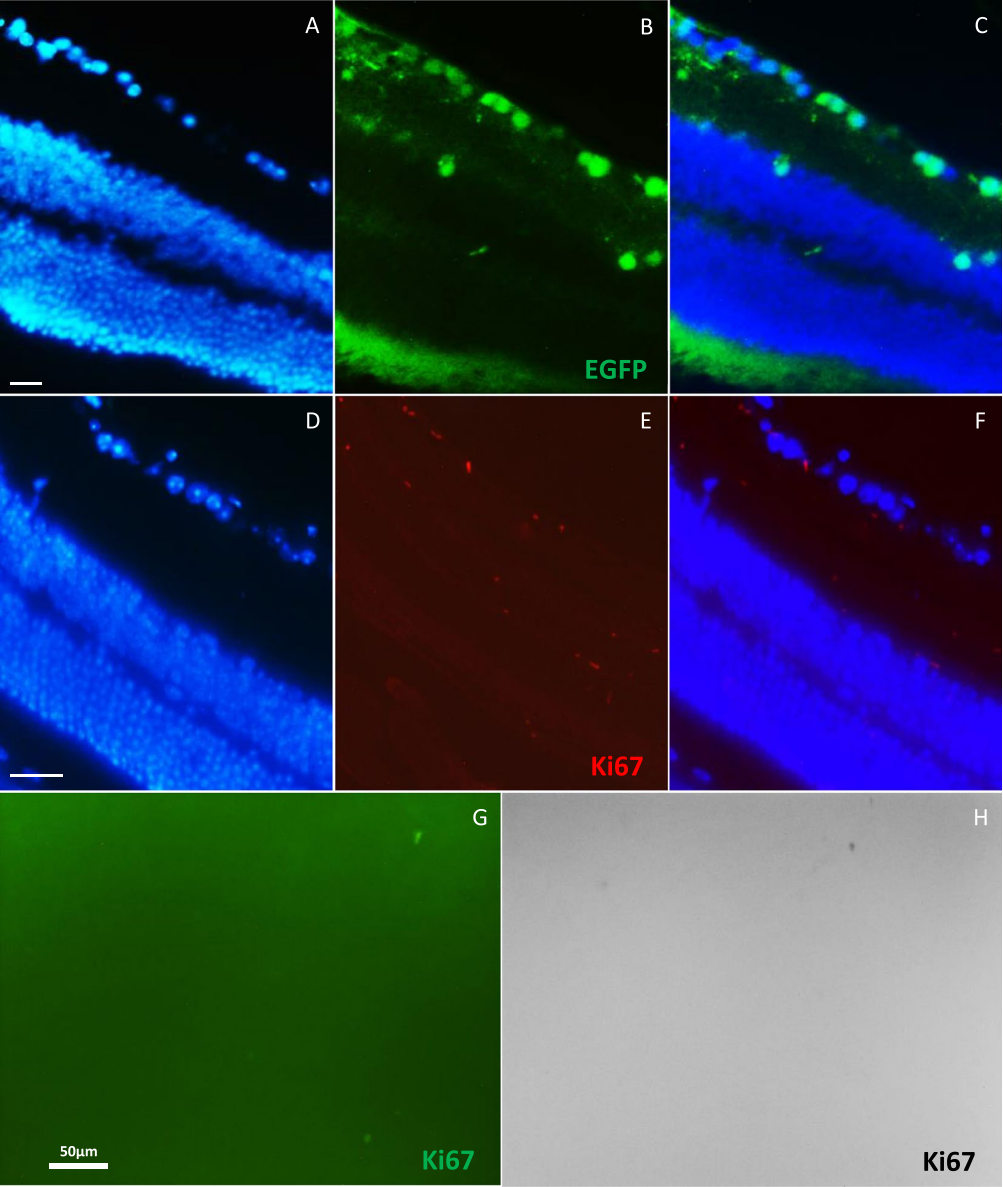
Analysis of Ki67 expression following Pax6 overexpression that was mediated by AAV2 viruses in normal mouse retina. (**A**–**C**) Immunofluorescence of EGFP, 30 days post Pax6 overexpression. EGFP was expressed in GCL layer and some inner INL cells. (**D**–**H**) Ki67 expression was not detected in infected normal retina in sections (**D**–**F**) and flatmount (**G**,**H**); H is black and white view of G with higher contrast. n > 5, scale bar: 50 µm.

**Figure 5:**
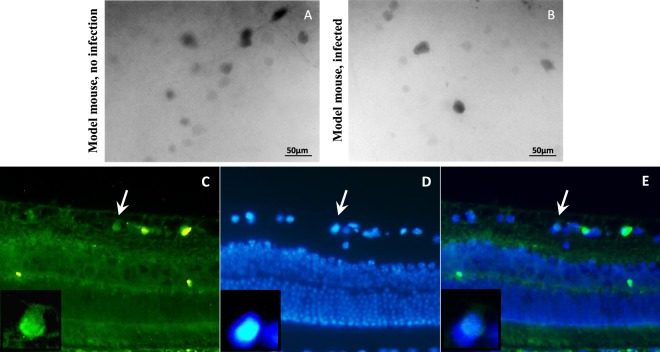
Analysis of Ki67 expression following Pax6 overexpression that was mediated by AAV2 viruses in model mouse retina. (**A**) Immunofluorescence of Ki67 in control flatmounts (only NMDA injection, no viruses), 30 days post infection; Ki67 was expressed obviously after NMDA injection. (**B**) Immunofluorescence of Ki67 in flatmounts, 30 days post NMDA and viruses injection; Ki67 was expressed as the same as the controls. (**C**–**E**) Immunostaining of Ki67 on 5 µm section following injections. Ki67 was expressed in GCL and some cells in INL.

We did not observe any changes in Ki67 expression following PAX6 overexpression in both of the model mice and its controls. In normal mice, Pax6 overexpression mediated by rAAV2-Pax6 viral particles could not induce Ki67 expression. In infected retina, EGFP expression was obvious (Fig. 4A–C), however we could not detected any Ki67 expression in retinal sections (Fig. 4D–F) or flatmounts (Fig. 4G,H). In model mice, Ki67 expression was significant increased, but it was the same for the control eyes. So, Ki67 expression was independent of Pax6 overexpression and was due to NMDA cytotoxicity (Fig. 5A–D).

### Evaluation of NMDA cytotoxicity in the mouse retina

**Figure 6:**
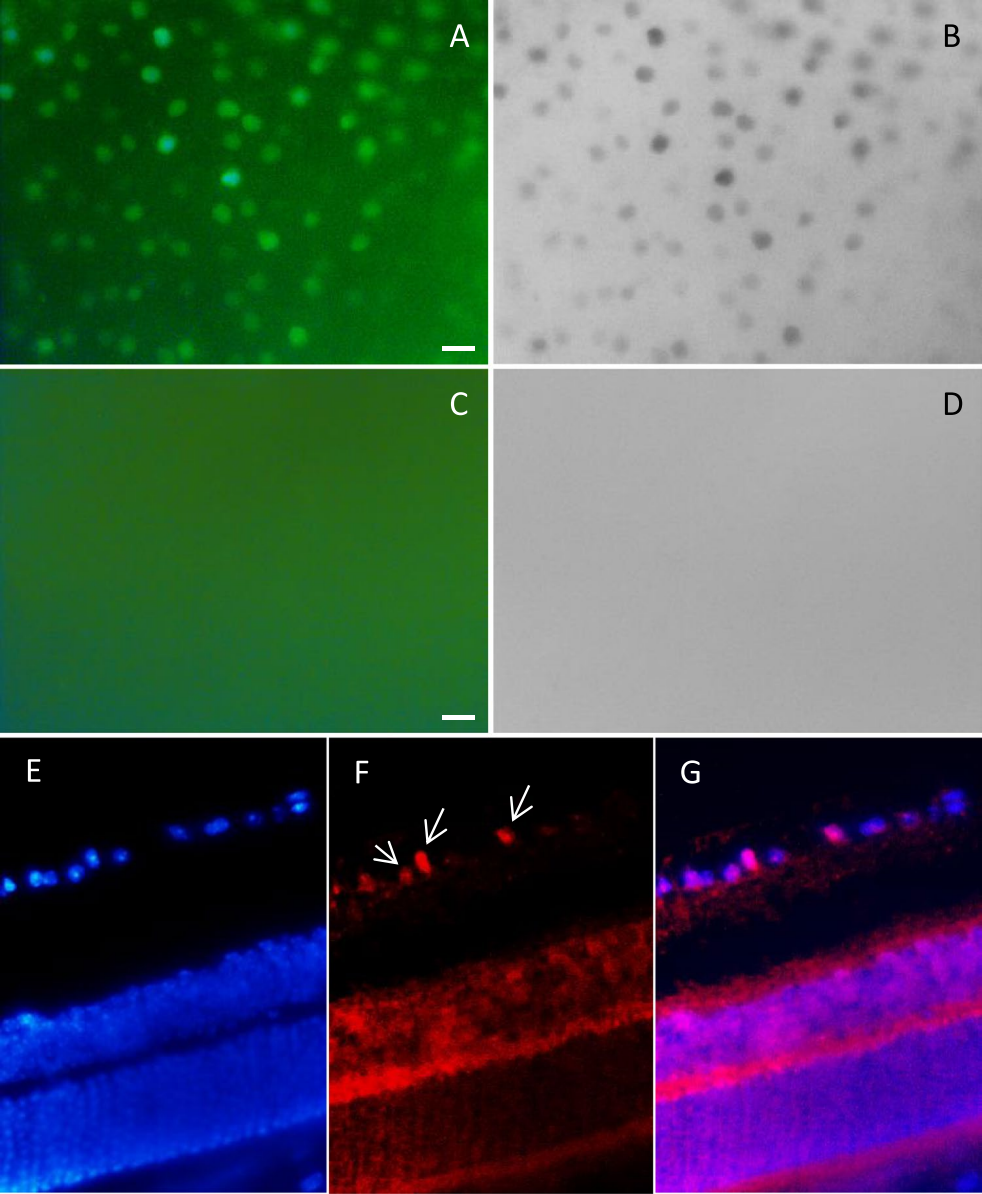
Immunofluorescence of Ki67, 3 months post NMDA injection. (**A**,**B**) Expression of Ki67 has been shown after 3 months on flatmounts (n = 2). (**C**,**D**) Flatmounts of vehicle injected retina as control. (**E**–**G**) Immunostaining of Ki67 on 5 µm section 3 months post NMDA injection. Ki67 was expressed in GCL (white arrows) and some cells in INL. Scale bar: 50 µm.

**Figure 7:**
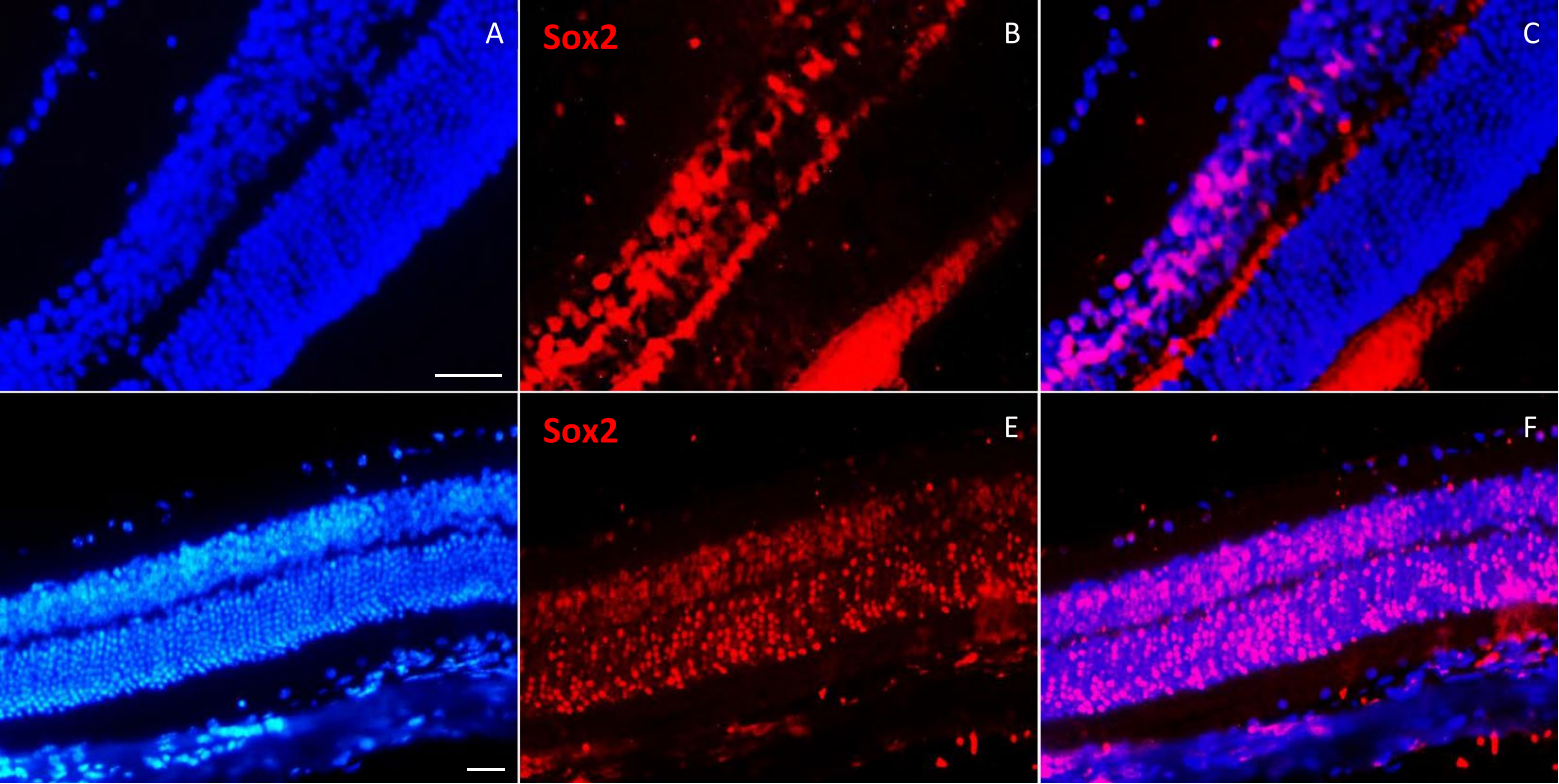
Immunostaining of Sox2 on normal and model mouse retina sections. (**A**–**C**) In normal mouse retina, Sox2 was expressed in some INL cells, but not in other retina layers. (**D**–**F**) In NMDA injected mouse retina (after 30 days), Sox2 was obviously expressed in ONL, INL and GCL layers. n > 3, scale bar: 50 µm.

NMDA is an excitotoxic amino acid that overstimulates NMDA-receptors and leads to cell death in RGC and amacrine cells. Our results showed significant Ki67 expression, in flatmounts, followed by NMDA injection in eyes. Also, IHC analysis on retinal sections clearly showed expression of Ki67 cell cycle marker in INL and GCL layers’ cells (Fig. 5C–E). We also followed Ki67 expression after three months of damage. Ki67 revealed sustained expression without significant reduction (Fig. 6). Interestingly, after 30 days of neural injury induction by NMDA, Sox2 expression was observed in ONL layer (Fig. 7). Sox2 is a stemness marker and its expression indicates neural plasticity of photoreceptors component of retina.

## Discussion

In recent years, one of the challenging fields in biology is using of intrinsic regeneration potential in damaged organs. In fact, when it was shown that with genetic or epigenetic manipulations we can reprogram differentiated cells to stem/progenitor state, it seems more reasonable to stimulate residing, naturally dependent, cells in damaged region to regenerate the desired cells. So, many studies have been directed to evaluate differentiation potential of a variety cell types and to investigate for new therapeutic approaches. Contrary to importance of retina in many ocular diseases, there are no satisfactory numbers of studies related to research of plasticity in retinal cell types. Parts of the regenerative mechanism in non-mammalian vertebrates remain in the mammalian retina and may provide a basis to develop new strategies in patients with retinal degenerations. It seems that our knowledge about differentiation and self-renewal potential of retinal cells and also the role of genetic and epigenetic factors is not pleasing. Here, we evaluated Pax6 overexpression and NMDA cytotoxicity to stimulate proliferation in the mouse retinal cells. We realized that PAX6, as a master transcription factor, would be a valid candidate in manipulation of GRN leading to a substantial regeneration response after excitotoxic cell death stimulation in RGC layer.

Despite, well established retina regeneration in cold-blood vertebrates^21^ and partially in avian^22,23^, we have a few documents of retina regeneration in mammalians. However, some groups have reported sufficiency of few cells in INL to re-enter the cell cycle^8,9^. Furthermore, there are successful reports on inducing regeneration in mouse and rat retina by transcription factors and/or growth factors^24–27^. In model mice, Ooto and colleagues showed few cells in INL, not in other layers, have remarkable potential in entrance to cell-cycle after neurotoxic damage with NMDA^8^. However, our results revealed some Ki67 positive cells in GCL in addition to robust Ki67 expression in INL. Retinal ganglion cells have significant roles in visual system^28^ and the most common retina diseases such as glaucoma^29^. Here, for the first time, we showed reentrance of GCL cells to the cell cycle after NMDA treatment. Suga and his colleagues showed the degree of proliferation rate in damaged cells of the retina varies between the mouse strains^7^, it suggests that, the difference in the intrinsic potency of regeneration between species is very important. This issue can fully affect results of different studies, technically and theoretically. We found a considerable plasticity in GCL cells and robust Ki67 expression, even, until three months after neurotoxic damage in INL following NMDA injection.

Pax6 is a pleiotropic transcription factor that has many challenging roles in retina developments. Here, we injected intravitreally AAV serotype 2 as a delivery vehicle of Pax6 to disturb GRN in retina cells. Up-regulation of some progenitor markers including Pax6, Notch and Dll1 has been reported after NMDA damage^9^. Here, we investigated whether Pax6 overexpression can promote proliferation in retina. We could not detect any changes in Ki67 expression following Pax6 overexpression. However we have to consider a few points; First, intravitreal injection of AAV2 yielded robust transgene expression throughout the retina in whole-mount analysis. Retinal sections showed the expression mainly resided in RGCs and some cells in INL layer as previously reported^30^. However, in normal adult retina, some INL cells and ganglion cells express Pax6 intrinsically; therefore, it is possible that Pax6 overexpression with non-integrative system was not sufficient to disturb GRN. Secondly, previous studies have been shown high autoregulation of Pax6 expression^31^. In fact, due to important roles of expression level of this transcription factor, cells have potent mechanisms to regulate Pax6. Thirdly, in recent studies, different roles of Pax6 isoforms are established^32,33^. Here, we recruited dominant isoform in the retina, 5a isoform, to our analyses.

An interesting finding in our data was the detection of Sox2 expression in ONL cells after neurotoxic injury. Sox2 has a key role in retina development and is one of the factors in iPS generation^10,34^. This transcription factor is expressed in retinal progenitors and, in adult mammalian retina, some INL cells, including muller glia cells^35^. Sox2 has a complex nature in interaction with main players in retina development such as Pax6^36^ and Wnt pathway^37^ and is a dose-dependent regulator of RPCs^38^. Previous studies in neural stem cells division demonstrated critical roles for Sox2; however there is limited knowledge about molecular mechanisms and chronic relationship in retina development. In *Xenopus* retina it has been demonstrated that Wnt signaling, mediated by Sox2, promotes neural destination by activating proneural gene expression and, coordinately, inhibit neural differentiation through Notch activation^39^. Here, we reported Sox2 expression in all retina layers including photoreceptors and GCL after 30 days of NMDA administration. Expression of Sox2 stemness marker promises high plasticity potential of retina cells and highlighted the role of microenvironment and extracellular signaling in acquisition of new phenotypes. More investigation is needed to analysis molecular pathways in retina and the cell fate machinery in reprogramming of retina cells.

## Conclusion

Reports on reactivation of inactive endogenous progenitors and well established retina regeneration in non-mammalians open up new hopes for using intrinsic potential of retinal cells in healing. It seems that we need new strategies in ocular degenerative therapies. Combination of different achievements in regenerative medicine introduces novel approaches to overcome limitations in cell and gene therapies. Entrance of retinal cells to cell cycle and expression of Sox2 stemness marker after neurotoxic damage along with origination of all retina cell types from one type of proliferative cells are a vivid prospect for retinal regenerative medicine.

## Materials and Methods

### Animals and care

All injections were performed on 6–8 week-old NMRI mice. Animals were housed in the National Institute of Genetic Engineering and Biotechnology (NIGEB) and all experiments were performed in accordance with the ARVO (Association for Research in Vision and Ophthalmology) statement for the use of animals and protocols in ophthalmic research that were approved by the ethical committee of; “Ophthalmic Research Center, Shahid Beheshti University of Medical Sciences” and “National Institute of Genetic Engineering and Biotechnology”. The mice were maintained under normal conditions of 12 h dark/light cycle, free access to water and food and 20–25 °C temperature.

### Plasmids and production of recombinant AAV virus

HEK293T cells were subjected to virus production and titration. PAX6a and IRES-EGFP (from pIRES2-EGFP) were cloned into pAAV-MCS (AAV Helper-Free System, Agilent). Triple transfection for two constructs (AAV-EGFP and AAV-PAX6/EGFP) was performed on twelve 10 cm plates by standard calcium-phosphate method. Briefly, the medium was changed 2 h before transfection. For each plates, 15 µg of each plasmids and 48 µl CaCl_2_ (2 M) reached to 400 µl final volume with ddH_2_O. This solution was added to 2× HEPES buffer saline (2× HBS: 595.7 mg HEPES, 13 mg Na_2_HPO_4_, 2H_2_O, 818.2 mg NaCl, 119 mg dextrose and 37 mg KCl in 50 ml ddH_2_O, pH 7.05) while vortexing. After 15 min incubation, DNA complexes were added to the cells. The medium was changed with 10% FBS fresh medium after 6 h. 68–72 hours later, viruses were purified and concentrated with HiTrap™ Heparin HP (GE Healthcare) and amicon columns (Millipore) as previously reported^40^. Viruses were aliquoted and stored at −80 °C. Viral titration was achieved with flow cytometry and qPCR. For flow cytometry, HEK293T cells were plated and infected according to standard protocol (Agilent tech., CA, USA). After 48 h, cultures were trypsinized and were washed twice with PBS. The cells were resuspended in PBS and GFP expression was analyzed (Cyflow space Partec, Germany, flowmax software). In qPCR, primers were designed for CMV promoter and standard curve were plotted. Genomic-based titration was done with Real-Time PCR.

### Intraocular Injections

All experiments were done in seven groups (1: no injected, 2: PBS injected as vehicle, 3: NMDA injected, 4: EGFP viruses injected, 5: Pax6/EGFP viruses injected, 6: EGFP viruses injected 48 h after NMDA, 7: Pax6/EGFP viruses injected 72 h after NMDA). For each treatment, right eye was its control and more than six animals were applied for each group. The eyes were injected with 80 µm glass needle connected to CellTram™ oil manual microinjector (Eppendorf). Mice were anesthetized by i.p. injection of 100 mg/kg ketamine and 10 mg/kg xylazine. Before injection, a drop of tropicamide (Mydrex 0.5%) and after injection, liposic ophthalmic gel (Bausch and Lomb) and gentamicin were administrated topically. Retinal damage was induced by the injection of 2 µl NMDA (20 mM, sigma) in PBS and for other analyses, 2 µl viruses or PBS were injected intravitreally. To confirm NMDA cytotoxicity, histological analyses were performed seven days after administration. Other analyses were done thirty days after injection.

### Histological Analysis of Mouse Retina

The mice were euthanized by cervical dislocation. The eyes were enucleated and after washing with PBS, fixed overnight in 4% paraformaldehyde. To evaluation of NMDA neurotoxicity, seven days after injection, three sections containing optic nerve and with maximal circumference of the eyeball were stained with hematoxylin and eosin (H and E). The number of ganglion cells and thickness of retinal layers were calculated and averaged in NMDA or PBS (vehicle) injected and normal (uninjected) eyes. To IHC analyses, the fixed tissues were washed in PBS and dehydrated in ethanol serials and xylene according to standard protocols and fixed in paraffin. 5 µm paraffin sections were subjected to IHC analyses. To whole-mount analysis, Iris and lens were removed and remained cup, fixed 10 min. in cold methanol. After three times washing, tissues were subjected to IHC. The antibodies and working dilutions in this study were as follows: mouse anti-GFP (sc-9996, 1:100), rabbit anti-Ki67 (sc-15402, 1:100), goat anti-Pax6 (sc-7750, 1:200), rabbit anti-PKC (sc-10800, 1:200), goat anti-Sox2 (sc-17320, 1:200), donkey anti-goat IgG-FITC (sc-2024, 1:200), goat anti-rabbit IgG-FITC (sc-2012, 1:200), goat anti-mouse IgG-FITC (sc-2010, 1:200), donkey anti-goat IgG-R (sc-2094, 1:100) and goat anti-rabbit IgG-R (sc-2091, 1:100).

### Imaging and Analyses

H and E stained sections were photographed by light microscope and the thickness of retinal layers analyzed at a distance of 350 µm or up to 1 mm in spider plots from the optic nerve. Also, ganglion cell counts were performed at a distance between 350 and 650 µm from optic nerve. The data were averaged and presented as ± S.E.M. (n = 3). Comparisons were done by one-way ANOVA (IBM SPSS Statistics 22) and P < 0.05 was considered significant. For IHC results, An Axiophot Zeiss fluorescence microscope equipped with a 520 nm filter for the FITC and a 460 nm filter for DAPI was used. For comparison of whole-mounts and making black and white images, ImageJ software was applied.

## Electronic supplementary material

Supplementary Figure 1
