# Supplementary material for: In Vivo Evaluation of PAX6 Overexpression and NMDA Cytotoxicity to Stimulate Proliferation in the Mouse Retina
Source: Sci Rep. 2018 Dec 7;8:17700. doi: 10.1038/s41598-018-35884-5 (PMC6286369; doi:10.1038/s41598-018-35884-5)
Supplement: Supplementary file 1 — Supplementary Figure 1 [file 41598_2018_35884_MOESM1_ESM.pdf]

In Vivo Evaluation of PAX6 Overexpression and NMDA Cytotoxicity to Stimulate Proliferation in the Mouse Retina

Ehsan Ranaei Pirmardan[a], Zahra-Soheila Soheili[b]*, Shahram Samiei[c], Hamid Ahmadieh[d], Seyed Javad Mowla[a], Marzieh Naseri[e], Narsis Daftarian[d]

[a] Department of Molecular Genetics, Faculty of Biological Sciences, Tarbiat Modares University, Tehran, Iran
[b] Department of Molecular Medicine, National Institute of Genetic Engineering and Biotechnology, Tehran, Iran
[c] Blood Transfusion Research Center, High Institute for Research and Education in Transfusion Medicine, Tehran, Iran
[d] Ophthalmic Research Center, Shahid Beheshti University of Medical Sciences, Tehran, Iran
[e] Department of Molecular Medicine, Faculty of Advanced Technology, Iran University of Medical Sciences, Tehran, Iran

* E-mail: soheili@nigeb.ac.ir, Tel: +989122990724

Supplemental Information

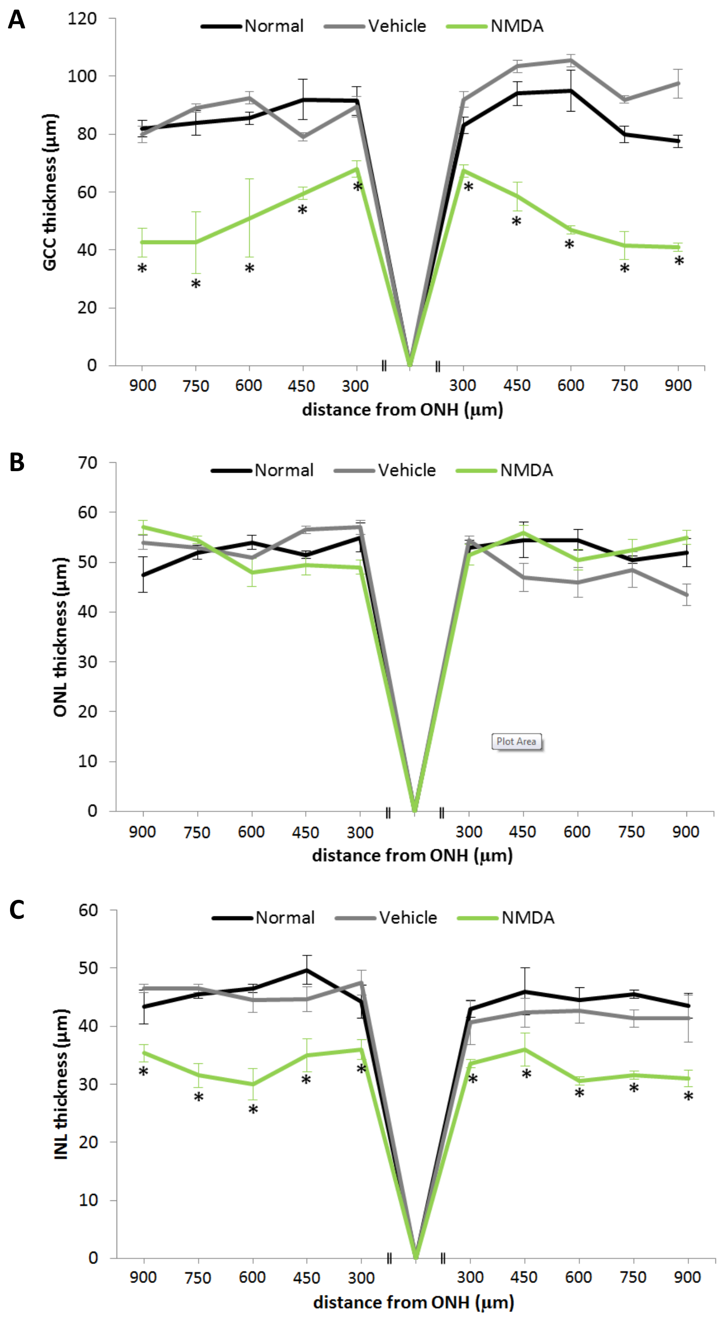

Supplementary Figure 1: Quantitative spider plot analysis of different layers of the retina. (A) Quantitative analysis of GCC thickness showed significant decrease in NMDA treated retina. (B) Regarding ONL thickness, there was no significant difference between groups. (C) Analysis of INL thickness showed significant decrease in NMDA group versus the other groups. (*, $P<0.05$ versus other groups, error bar: means ± SD).
